# Compaction of rolling circle amplification products increases signal integrity and signal-to-noise ratio

**DOI:** 10.1038/srep12317

**Published:** 2015-07-23

**Authors:** Carl-Magnus Clausson, Linda Arngården, Omer Ishaq, Axel Klaesson, Malte Kühnemund, Karin Grannas, Björn Koos, Xiaoyan Qian, Petter Ranefall, Tomasz Krzywkowski, Hjalmar Brismar, Mats Nilsson, Carolina Wählby, Ola Söderberg

**Affiliations:** 1 Department of Immunology, Genetics and Pathology, Uppsala University, Science for Life Laboratory, Biomedical center, SE-75108 Uppsala, Sweden; 2 Department of Information Technology, Centre for Image Analysis, Uppsala University, Science for Life Laboratory, SE-75105 Uppsala, Sweden; 3 Department of Biochemistry and biophysics, Stockholm University, Science for Life Laboratory, Svante Arrhenius väg 16C, SE-10691 Stockholm, Sweden; 4 Science for Life Laboratory, Royal Institute of Technology, Box 1031, SE-171 21 Solna, Sweden

## Abstract

Rolling circle amplification (RCA) for generation of distinct fluorescent signals *in situ* relies upon the self-collapsing properties of single-stranded DNA in commonly used RCA-based methods. By introducing a cross-hybridizing DNA oligonucleotide during rolling circle amplification, we demonstrate that the fluorophore-labeled RCA products (RCPs) become smaller. The reduced size of RCPs increases the local concentration of fluorophores and as a result, the signal intensity increases together with the signal-to-noise ratio. Furthermore, we have found that RCPs sometimes tend to disintegrate and may be recorded as several RCPs, a trait that is prevented with our cross-hybridizing DNA oligonucleotide. These effects generated by compaction of RCPs improve accuracy of visual as well as automated *in situ* analysis for RCA based methods, such as proximity ligation assays (PLA) and padlock probes.

A rolling circle amplification product (RCP) is a long repetitive single-stranded amplicon consisting of hundreds of reverse complementary elements of a circular template, lined up in a single molecule^1^. These RCPs can be probed with fluorophore labeled oligonucleotides, i.e. detection oligonucleotides, to visualize single molecules *in situ* by standard fluorescence microscopy. Several methods rely on rolling circle amplification (RCA) to generate signals *in situ*, such as immuno-RCA (iRCA)^2^, Proximity Ligation Assay (PLA)^3^ and padlock probes^4^,^5^. Immuno-RCA is used to detect a single protein by an antibody conjugated to an oligonucleotide. To this a circular DNA template can be hybridized and then amplified by the enzyme phi29 polymerase, using the oligonucleotide conjugated to the antibody as a primer. This will create a RCP, covalently linked to the antibody. In PLA, two antibodies are conjugated with two different oligonucleotides (proximity probes). If the proximity probes bind to their targets in close proximity they will act as a hybridization/ligation template for two additional oligonucleotides and a circular DNA template can be created by ligation, which can be amplified using one of the proximity probes as a primer for RCA. Padlock probes are used to detect DNA or mRNA and are sensitive enough to detect single nucleotide variations. The padlock probe consists of an oligonucleotide with its ends complementary to its target sequence (DNA or cDNA). Upon hybridization of the padlock to the target a circular template is created by ligation of the juxtaposed two ends of the padlock probe. The DNA, or cDNA sequence will be used as a primer for RCA.

It has been thought that an RCP collapse onto itself by the polarity inherent to DNA, to fold into a random coil conformation^6^. However, we have found that some RCPs are poorly pulled together, and tend to split up into clusters, easily mistaken for several signals.

An obstacle with RCA-based methods is that with an increased concentration of RCPs, the RCPs start to coalesce and individual products cannot be discerned^7^. Therefore, it is desirable to develop RCA based assays that can generate signals smaller in size for an increased dynamic range.

We herein present a novel approach to generate smaller signals with higher signal intensity, better signal-to-noise ratio and improved integrity, by including an oligonucleotide that generates more compact RCPs. These properties facilitate a more accurate image analysis for RCA-based methods.

## Results

### The compaction oligonucleotide design

**Figure 1:**
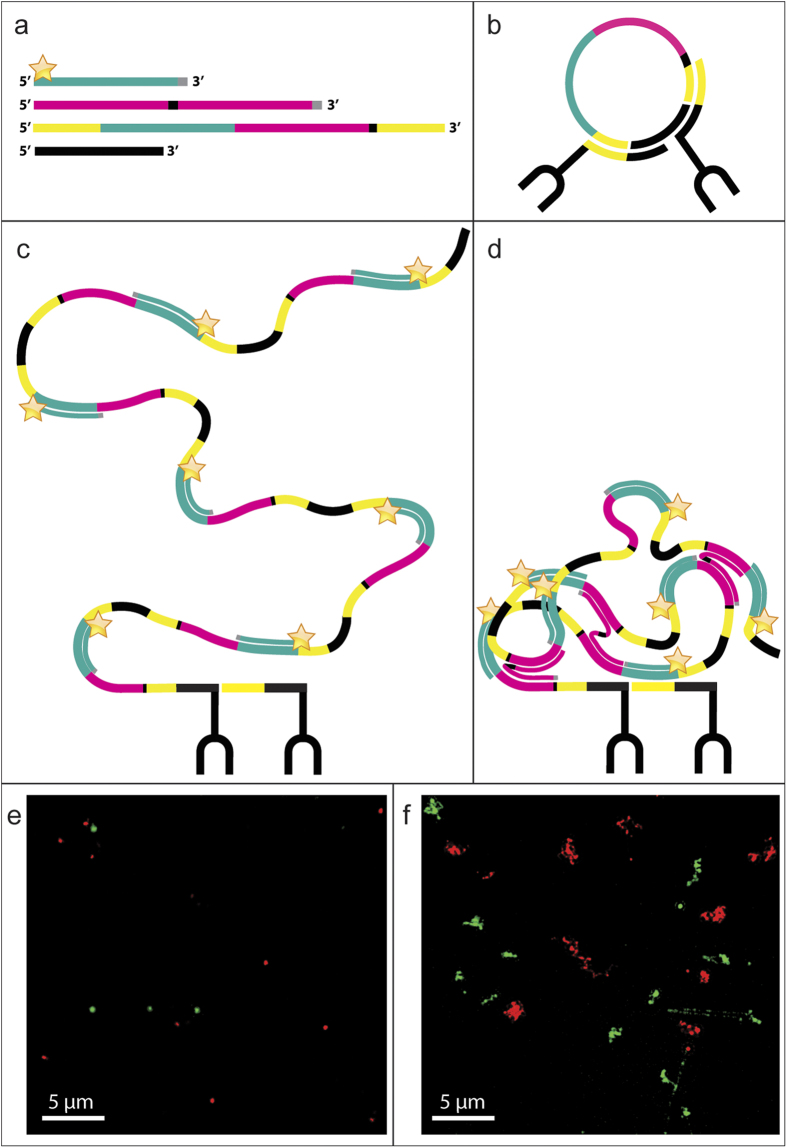
Schematic cartoon and visualisation of regular vs. compacted RCPs. The oligonucleotides from top to bottom in (**a**) are the detection oligonucleotide, the compaction oligonucleotide, the long circularization oligonucleotide and the short circularization oligonucleotide. The detection oligonucleotide (Table 1, oligonucleotide **A**) has the same sequence as a part of the long circularization oligonucleotide (cyan), nucleotides preventing degradation and priming (grey) by the polymerase and a fluorophore (star). The compaction oligonucleotide (Table 1, oligonucleotide **B**) has two copies of the same sequence also found in the long circularization oligonucleotide (magenta), a spacer sequence (black) and nucleotides preventing degradation and priming (grey). Apart from the already mentioned sequences, the long circularization oligonucleotide (Table 1, oligonucleotide **C**) also has a spacer sequence (black) and parts hybridizing to the PLA probes (yellow). The short circularization oligonucleotide (oligonucleotide **D** in Table 1) has a sequence complementary to each PLA probe (not colored), spaced apart by a short sequence (not colored). The circularization oligonucleotides are ligated together in a separate step preceding the RCA reaction, resulting in (**b**). In regular RCA, the fluorophore labelled detection oligonucleotide is the only added oligonucleotide and the resulting RCP is depicted in (**c**). Adding also the compaction oligonucleotide to the RCA reaction results in a less dispersed RCA product (**d**). The bottom images were acquired with a 3D Structured Illumination Microscope and depict RCPs generated from two different circular templates with the dissimilarity of two different sequences for detection oligonucleotide hybridization. One of the detection oligonucleotides labelled with Alexa488 (green) and the other with Alexa642 (red). The images show RCPs with (**e**) and without (**f**) compaction oligonucleotide.

An RCP is a concatenation of multiple copies of the same sequence, several hundred can be present in the same molecule. Each copy of the RCP contains parts used for hybridization to fluorophore-labeled detection oligonucleotides. As an attempt to reduce the size of the RCPs we designed a cross-hybridizing probe (compaction oligonucleotide), which is composed of two identical sequences with each copy reverse-complementary to a part of each repeat in the RCP. The compaction oligonucleotide is 48 nucleotides long, containing two elements of 21 nucleotides for hybridization to the RCPs, which are separated by a spacer of three nucleotides. There are three nucleotides of 2'O-methyl-RNA at the 3' end to prevent degradation by the phi29 polymerase (Table 1). This enables the compaction oligonucleotide to pull both close and distal parts of the RCPs together via oligonucleotide hybridization (Fig. 1a–d).

### Signal size

In order to achieve a well-controlled environment for the analysis of RCPs, we used padlock probes that hybridized to a synthetically designed biotinylated template oligonucleotide anchored directly on streptavidin coated microscopy slides. Upon hybridization of the padlock probes to the template the gap between the 3' and 5' end of the padlock probe were ligated by T4 ligase and the created circular template amplified by Phi29 polymerase to generate RCPs. For image acquisition we used a 3D Structured Illumination Microscope (SIM) with a resolution of <80 nm, to be compared to a regular epifluorescence microscope with a resolution of above 200 nm. The result from adding the compaction oligonucleotide to the RCA reaction provides substantially more compact RCPs with a reduced diameter as compared to regular RCPs. Dual colored RCPs were generated to make sure that the regular RCPs are generated from one individual circularized templates. The circularized templates of the differently colored RCPs use the same sequence for target hybridization, but different sequences for hybridization of the fluorophore-labeled detection oligonucleotides (Fig. 1e,f). No color overlap occurred in either compacted or regular RCPs, which indicates that each signal in the images of regular RCPs originate from a unique amplification event. The effect of compaction is less pronounced when the oligonucleotide is added after rather than during RCA (data not shown).

### Signal intensity and signal-to-noise ratio

**Figure 2:**
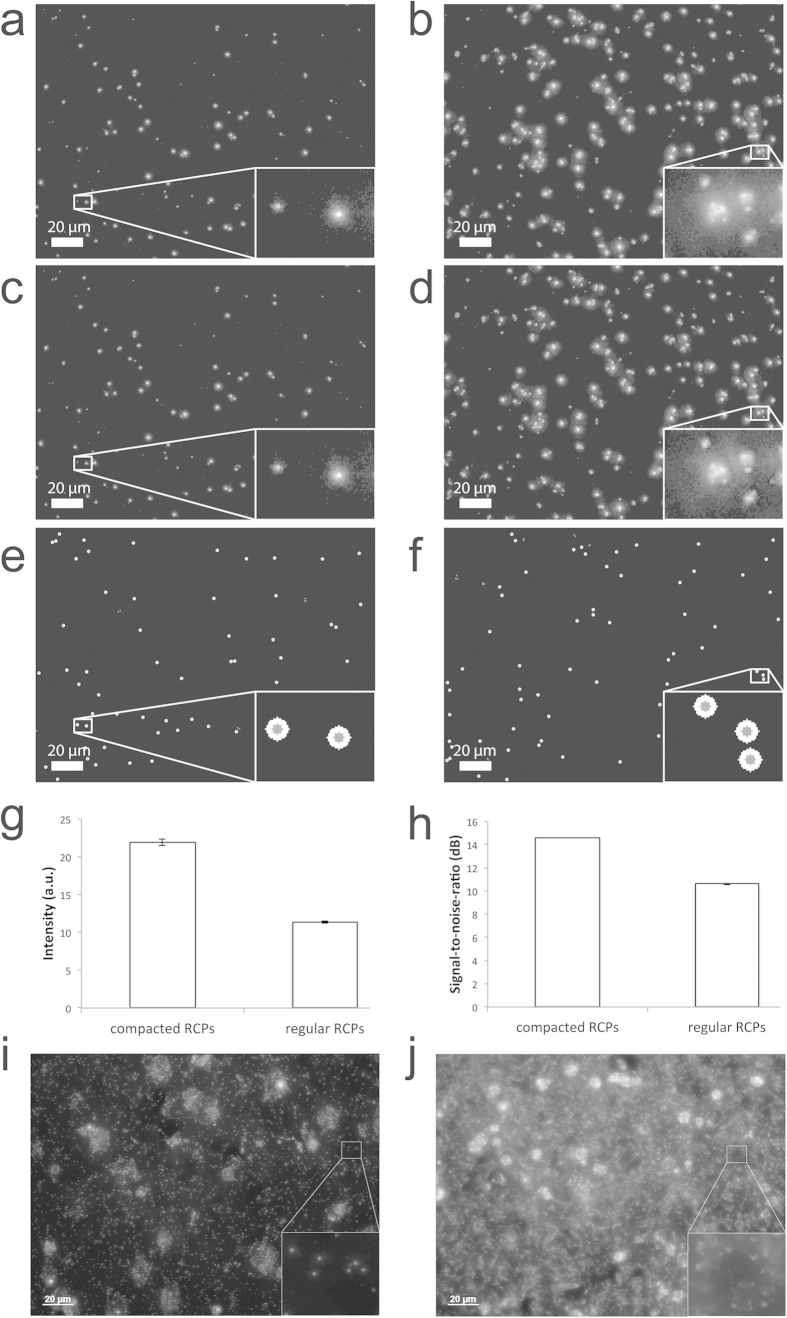
Comparison of signal intensity and SNR of regular vs. compacted RCPs. Compacted RCPs (**a**, **c**, **e**) are compared to regular RCPs (**b**, **d**, **f**) throughout steps in the image analysis procedure. Maximum projections of the z-stacks (**a**, **b**) are background corrected (**c**, **d**) before segmentation of signals (**e**, **f**). The detected signals are shown as gray disks and the signals used for signal-to-noise ratio measurements have white rings after segmentation. The white rings designate the local area within which noise is measured. The resulting intensity values (**g**) and signal-to-noise ratio (**h**) are depicted as bar plots. Significantly brighter signals arise from compacted RCPs (21.9 intensity units; s.e.m. 0.39) as compared to regular RCPs (11.4 intensity units; s.e.m. 0.16) p < 0.001 [Mann-Whitney U test]). The signal-to-noise ratio for compacted RCPs was significantly higher at 14.6 dB (s.e.m. 0.03) as compared to 10.6 dB for regular RCPs (s.e.m. 0.05) p < 0.001 [Mann-Whitney U test]). Error bars are standard errors of the means. PLA was used with and without the compaction oligonucleotide, to detect progranulin in hippocampal tissue. Compacted RCPs (**i**) are visually compared to regular RCPs (**j**).

RCP compaction results in a visually higher intensity, due to increased local concentration of fluorophore-labeled detection oligonucleotides (Fig. 2a vs b). We quantified the increase in local fluorescence of RCPs generated on biotinylated oligonucleotides anchored on streptavidin coated microscopy slides by automated measurement of signal intensities from maximum projections of the z-stacks (Fig. 2a vs b). We corrected for background variation (Fig. 2c vs d) before detection and delineation of signals (Fig. 2e vs f). Significantly brighter signals arise from compacted RCPs (21.9 intensity units; SD = 11.8 intensity units, representative experiment of n = 3 ) as compared to regular RCPs (11.4 intensity units; SD = 6.3 intensity units, corresponding experiment of n = 3), p < 0.001 [Mann-Whitney U test] (Fig. 2g). Compacted RCPs also appear less spread out than regular RCPs (Fig. 2a vs b). We quantified this spread by comparing the intensity of isolated signals in a circular inner disk of radius 3 pixels (i.e., gray disk in Fig. 2e,f) with the local background intensity variation measured in an outer disk of radius 7 pixels (i.e., white disk in Fig. 2e,f), thus approximating the signal to noise ratio (SNR). Tightly clustered signals were excluded as these make it difficult to distinguish between signal and local background, as described in the Material and Methods section. Signal-to-noise ratio (SNR) was converted to decibel (dB) by SNR = 20 × log_10_(*r*). The SNR for compacted RCPs was significantly higher (14.6 dB; SD = 2.0, representative experiment of n = 3) as compared to regular RCPs (10.6 dB; SD = 3.0, corresponding experiment of n = 3); p < 0.001 [Mann-Whitney U test] (Fig. 2h). In order to be sure that our manual choice of inner and outer-disk radii size (Fig. 2e,f) does not affect the result, two different radii (i.e., (3,7) and (5,12) pixels) of the inner and outer-disk were tested. Varying the size of the inner and outer-disk radii did not change the fact that compacted RCPs have a higher statistical significant signal intensity and SNR than regular RCPs (see Supplementary Table S1 online).

While observing better defined signals of compacted RCPs compared to regular RCPs *in vitro* on microscopy slides, we decided to also compare compacted RCPs to regular RCPs *in situ* in consecutive human hippocampal tissue sections, using *in situ* PLA for progranulin detection (Fig. 2i,j). A diffuse staining is visible between regular RCPs, while the pixels between compacted RCPs have lower intensity. We propose that the diffuse staining arise from parts of the RCP that diffuse out in the surrounding from the main RCP bundle (Fig. 1e,f). In areas where no RCPs are present the background intensity is the same for compacted and regular RCPs. The compacted RCPs are smaller and easier to distinguish from each other, as well as from the background fluorescence of the tissue section.

### Signal integrity

**Figure 3:**
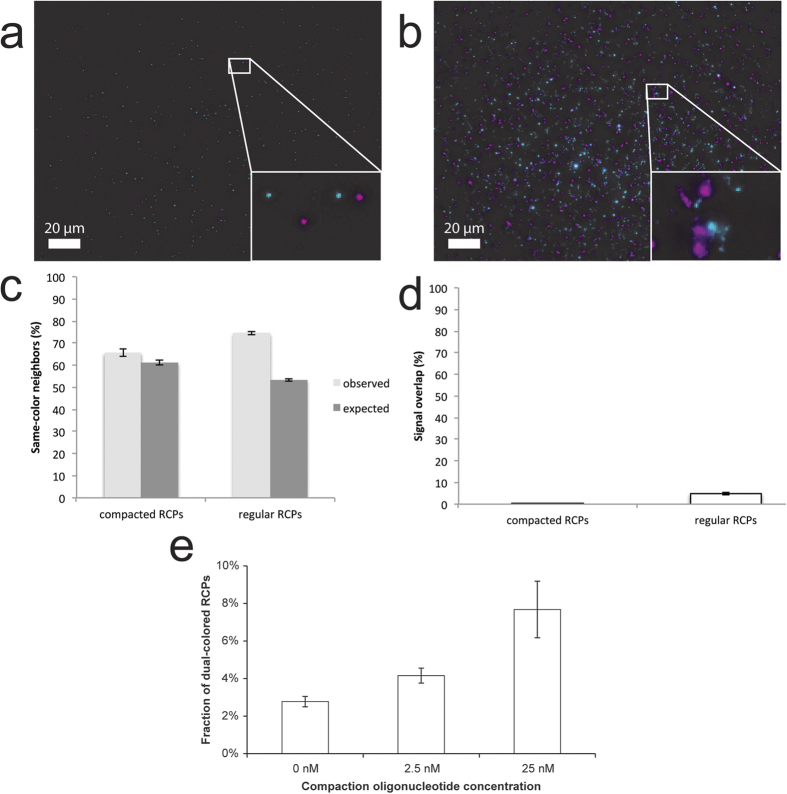
Signal integrity of regular vs. compacted RCPs. Compacted RCPs (**a**) are compared to regular RCPs (**b**), produced in a spatially random distribution from two different circular templates giving rise to RCPs appearing in two different wavelengths (pseudo-colored as magenta and cyan). For compacted RCA, the frequency of same-color neighbors is 65.6% (**c**; s.e.m. 1.8%) which is within the expected frequency as determined by Monte Carlo simulation (61.2%; s.e.m. 1.1%). For regular RCA, the frequency is 74.5% (s.e.m. 0.7%) which is significantly more than expected by Monte Carlo simulation (53.3%; s.e.m. 0.5%; p < 0.001, two tailed t-test)). Looking at the signal overlap (**d**), this shrinks to 0.23% (**d**; s.e.m. 0.07 ) by application of the compaction oligonucleotide. The overlap turned out to be 4.78% (s.e.m. 0.49%) for regular RCPs. The two groups are significantly different in signal overlap (p < 0.001 [two-tailed t-test]). Error bars are standard errors of the means. To study the effects of the new oligonucleotide design in solution, two types of RCPs labelled with either Cy3 or Cy5 were produced together with 0, 2.5 or 25 nM of compaction oligonucleotide in solution (**3e**). The fraction of dual-colored RCPs was determined to depend upon the concentration of the compaction oligonucleotide (p = 0.022 [ANOVA]). The fraction of dual-colored RCPs was not significantly different between 0 and 2.5 nM of the compaction oligonucleotide (p = 0.326 [ANOVA with LSD post hoc test]), but a significant difference was found between 0 and 25 nM (p = 0.009 [ANOVA with LSD post hoc test]), and between 2.5 and 25 nM (p = 0.034 [ANOVA with LSD post hoc test].) The fraction of dual-colored RCPs at 0 nM of the compaction oligonucleotide is 2.78% (s.e.m. 0.28%, n = 3, total of 63,795 signals) for 2.5 nM it is 4.16% (s.e.m. 0.69%, n = 3, total of 43,542 signals) and for 25 nM it is 7.68% (s.e.m. 2.61%, n = 3, 14,019 signals). Error bars are standard errors of the means.

The images obtained by SIM showed that regular RCPs are sometimes dispersed into several foci of higher fluorescence (Fig. 1e,f), which was not the case for compacted RCPs. This prompted us to investigate whether this could be detected using regular epifluorescence microscopy. To quantify the integrity of the RCPs with and without a compaction oligonucleotide, we took the same approach as used for SIM, i.e. RCPs reporting in two different colors in a spatially random distribution over a microscopy slide (Fig. 3a,b). The experiment was performed in three replicates. We investigated the difference between regular and compacted RCPs by measuring the fraction of neighboring RCPs with the same color. If an RCP splits up into two or more signals, they will be located close together and be of the same color. We hypothesized that the compacted RCPs generate signals with higher integrity and thus have a lower probability of forming signal clusters of the same color. The hypothesis was tested by calculating the ratio of signals with the same color closest neighbor to the total count of signal pairs. As per our hypothesis, this ratio should be lower for compacted RCPs and higher for regular ones. This ratio was compared to a Monte-Carlo probability distribution method. The method was used to simulate the probability of an RCP having a closest neighboring RCP of the same color, if the RCPs (occurring in two different colors) are randomly distributed within a dataset with fixed occurrence frequencies. Monte Carlo simulations were performed ten times for each of three replicated experiments. For each replicate, the simulated results were combined into a mean value, which was used for comparison with the experimental result of the corresponding replicate. For compacted RCPs the same color neighbors occurred at a frequency of 65.6% (SD = 6.6%, one experiment of n = 3, a total of 18,563 magenta and 6,680 cyan signals), which is within the margins of the expected random frequency of 61.2% generated by a Monte-Carlo simulation (SD = 4.0%; p = 0.053, two-tailed t-test). For RCPs without compaction oligonucleotide, the same color neighbors occurred at a frequency of 74.5% (SD = 2.4%, corresponding experiment of n = 3, a total of 45,352 magenta and 26,004 cyan signals, Fig. 3c), which is significantly higher than the expected random frequency of 53.3% (SD = 1.6%; p < 0.001, two tailed t-test), thus supporting our hypothesis. When looking at all three experiments we find that two out of three do not show significant differences between compacted RCPs and the corresponding Monte Carlo simulation. One experiment showed significant differences between the two distributions (p < 0.001, two tailed t-test). However the relative difference between the two mean values was decidedly smaller (3.2%) for this experiment than for any of the regular RCP experiments (difference between regular RCPs and their respective Monte Carlo simulation ranged from 12.9% to 21.2%; all three experiments p < 0.001, two tailed t-test). Therefore we feel confident to conclude that compaction oligonucleotides do indeed greatly reduce dispersion of RCPs. Furthermore, we hypothesized that dispersed signals are more likely to result in signal overlap, and therefore a larger proportion of dual-colored pixels. We used the same images as above to study the percentage of signal overlap between the two colors. Compacted RCPs had 0.23% signal overlap (SD = 0.26%, representative experiment of n = 3) and regular RCPs had 4.78% signal overlap (SD = 1.77%, corresponding experiment of n = 3, Fig. 3d). The two groups were found to be significantly different (p < 0.001 [Mann-Whitney U test]), indicating that regular RCPs are more dispersed than compacted RCPs. Low signal overlap of compacted RCPs also indicates that the compaction oligonucleotide does not cross-link and aggregate neighboring anchored RCPs.

### RCP aggregation in solution

In order to determine whether compaction oligonucleotides can cause RCPs generated in solution to aggregate, we simultaneously amplified 2 types of DNA circles containing the same target sequence but different sequences for detection (i.e. Cy3- and Cy5-labeled detection oligonucleotides) in presence with and without compaction oligonucleotide. For image acquisition, RCPs were flowed through a microfluidic channel and time line scan images were acquired using a confocal microscope, as described in detail elsewhere^8^. We investigated the extent of RCP aggregation at varying concentrations of compaction oligonucleotide by measuring the overlap of Cy3 and Cy5 fluorescence within RCPs (Fig. 3e). At a set concentration of 10 pM of the circular template, we varied the compaction oligonucleotide concentration between 0 nM, 2.5 nM and 25 nM during RCA, to study if signal overlap is influenced by increased compaction oligonucleotide concentration. There was no significant change of signal overlap between 0 nM and 2.5 nM of compaction oligonucleotide (p = 0.326 [ANOVA with LSD post hoc test]), but a significant change of signal overlap is apparent when increasing the compaction oligonucleotide concentration from 0 nM to 25 nM (p = 0.009 [ANOVA with LSD post hoc test]) and also from 2.5 nM to 25 nM (p = 0.034 [ANOVA with LSD post hoc test].). These findings indicate cross-linking of RCPs when RCA is performed in solution in presence of elevated concentrations of compaction oligonucleotides.

## Discussion

The RCA modification presented in this paper has several benefits for *in situ* assays employing RCA for signal generation. Addition of the compaction oligonucleotide to the RCA makes the RCPs smaller and more compact. The local concentration of fluorophores increases with compaction of the RCP, resulting in higher fluorescence intensity and an increased SNR, which is particularly useful in samples that typically suffer from high autofluorescence. These more compact and intense signals also facilitate precise enumeration of the signals both visually and by automated image analysis.

An additional benefit of this modified RCA technique may be the prevention of multiplication of signals appearing in regular RCA. We have previously expected the single-stranded DNA (ssDNA) product arising from regular RCA to collapse onto itself via the inherent polarity of ssDNA and the random hydrogen bonding of the bases, to create a compound ssDNA bundle. However, regular RCPs are not always strongly pulled together, and we have herein shown that several closely residing fluorescence foci may originate from the same RCP, probably due to translocation of the nascent strand during polymerization. Compaction does increase the integrity of RCPs and reduce the amount of dispersed signals, which will give a more accurate signal representation of the investigated target.

Not all areas of RCA application benefit from compaction. For instance, the quantification of RCA products generated in solution would likely be hampered by compaction-RCA, as the compaction oligonucleotides cross-link individual RCA products. The RCA has to start from an anchored DNA strand, i.e. genomic DNA or a DNA-strand conjugated to an antibody anchored to its antigen, in order to avoid clustering of several individual RCPs.

The improvements that compaction of RCPs provide facilitate a more precise signal representation and quantification. The compaction of RCPs can be directly applied for several RCA-based methods, with a minimal practical effort of just adding an extra oligonucleotide to the RCA reaction.

## Materials and Methods

### RCA on slides

We performed RCA directly on microscopy slides in order to provide a better-controlled environment for image analysis of the RCPs. We did all washes twice for 2 min in TBS unless stated otherwise. Streptavidin-coated TRIDIA BA Slides (SurModics IVD Inc.) with Secure Seals (Life Technologies), each holding a volume of 40 μl was used. We distributed 0.5 nM (for signal intensity, SNR, and signal integrity experiments) or 10 μM (for the SIM visualization experiment) of a biotinylated RCA anchor (Table 1, oligonucleotide E) diluted in Tris-buffered saline (TBS), into each well, and incubated at room temperature for 10 min. We washed and added a hybridization mix containing 0.01 μM pre-mix of two long circularization oligonucleotides (named oligonucleotides F and G), the short circularization oligonucleotide (named oligonucleotide D) and the ligation template (named oligonucleotide H) plus 0.25 mg ml^–1^ BSA (New England Biolabs), 250 mM NaCl, 0.05% (vol/vol) Tween-20 (Sigma-Aldrich), 10 mM TrisAc, 10 mM MgAc and 50 mM KAc, pH 7.5, followed by incubation for 30 min at 37°C. After washing we ligated the circularization oligonucleotides with a ligation reagents mixture which was the same as the aforementioned hybridization mixture apart from the absence of the oligonucleotides and the presence of 0.5 mM ATP and 0.025 u μl^–1^ of T4 ligase. The reaction took place for 15 min at 37 °C before washing. We performed the RCA both with and without 25 nM of the compaction oligonucleotide (Table 1, oligonucleotide B). The RCA took place in the presence of 25 nM of detection oligonucleotides (oligonucleotides I and J for signal intensity, SNR, and signal integrity experiments) oligonucleotides L and Q for the SIM visualization experiment) in a RCA buffer containing 0.25 mg ml^–1^ BSA, 7.5 ng ml^–1^ poly-adenosine, 1x phi29 DNA polymerase buffer (Fermentas), 0.25 mM dNTP (Thermo Scientific) and 0.25 U μl^–1^ phi29 DNA polymerase, followed by incubation at 37 °C for 1 h. After washing we removed the secure seal and dehydrated the slide by washing with an EtOH solution (70% (vol/vol) EtOH and 30% (vol/vol) TBS) for 2 min and secondly with a 99% (vol/vol) solution of EtOH for 2 min. We then centrifuged the slide for 10 s and mounted with SlowFade.

### Tissue slide preparation and PLA

We did all washes twice for 2 min in TBS unless stated otherwise. We used two 4 μm thick consecutive sections of anonymized formalin-fixed and paraffin-embedded hippocampal tissues from post-mortem brain of non-demented elderly cases, purchased from the Netherlands Brain Bank. We removed paraffin by serial washes in xylene and rehydrated in an ethanol series, and then performed antigen retrieval in pH 6.0 citrate buffer (Dako Denmark A/S), heated to 125 °C for 5 min, followed by 80 °C for 20 min. An ImmEdge™ Pen (Vector Laboratories) was used to create hydrophobic perimeters around the tissue specimens. We blocked each sample with blocking solution (Olink Bioscience AB) for 1 h at 37 °C and continued by incubating the samples with goat-derived antibody to progranulin at a concentration of 3.33 μg ml^–1^ over night at 4 °C. We washed and incubated the samples with species-specific secondary proximity probes (Olink Bioscience AB) diluted 1:5 in Olink Antibody dilution buffer (Olink Bioscience AB) for 1 h at 37 °C. After a wash, we hybridized 125 nM of a long and a short circularization DNA oligonucleotide, named C and D to the proximity probe arms and incubation conditions as above. The slides were washed and the ligation of the circularization oligonucleotides was performed in the same ligation buffer and conditions as above. Samples were washed and the RCA buffer was the same as above but with 1 μM Hoechst 33342 (Fluka Biochem), 0.025 μM of detection oligonucleotides A plus 0.025 μM of compaction oligonucleotide (oligonucleotide B) where applicable (see results). We incubated the RCA and detection step at 37 °C for 1 h after which we washed, and additionally washed twice for 2 min with only TBS. Drying and mounting of slides were performed as above.

### RCA in solution

We first hybridized 10 nM of two distinct long circularization oligonucleotides (oligonucleotide G and F) with 40 nM of ligation template (Table 1, oligonucleotide O) in two separate reactions in the hybridization buffer used above with the addition of 0.025 U μl^−1^ T4 DNA ligase (Fermentas) and 0.5 mM ATP (Fermantas), followed by 30 min incubation at 37 °C. We combined the two mixtures into one and preformed RCA with 0.01 nM of circular templates together with either 0 nM, 2.5 nM or 25 nM of compaction oligonucleotide (Table 1, oligonucleotide B). We did this in the RCA buffer described above, also including 5 nM of two detection oligonucleotides labeled with either Cy3 or Cy5 (oligonucleotides I and P), followed by incubation at 37 °C for 60 min before we put it on ice to stop the reaction. We enumerated the RCPs as described below in Confocal microscopy of RCPs in solution.

### Equipment and settings

#### Manual epifluorescence microscopy

For image acquisition we used a Zeiss Axioplan 2 imaging microscope equipped with filters optimized for each of the fluorophores, a 40 × (1.3 NA) objective and a Zeiss AxioCam MRm camera giving 1,388 × 1,040 pixel images (0.48 μm per pixel, 14 bit). The exposure times were kept the same for all samples within each experiment. For the tissue experiments we acquired 16 z-levels 462 nm apart of each area and for the RCA. On microscopy slides we acquired 12 z-levels with 800 nm in between. For both datasets we chose the sites for acquisition randomly. We manually adjusted contrast, brightness and gamma in the images (to the same degree for both controls and live experiments) that are presented in figures for visualization purposes, while we used raw images for image analysis.

#### 3D Structured Illumination Microscopy (3D-SIM)

We used 3D-SIM to produce high-resolution visual representations of RCPs on microscopy slides, while regular epifluorescence microscopy was used otherwise. We performed 3D-SIM^8^ imaging using a Plan-apochromat 100 × (1.46 NA) oil objective on an ELYRA PS.1 (Carl Zeiss) microscope. Excitation wavelengths were 488 nm with detection at 495–575 nm and 642 nm with detection at long-pass 655 nm. Images of 1,904 × 1,900 pixels with a pixel size of 25 nm were acquired in 5 rotations and 5 phases. We reconstructed the final images using the SIM module in ZEN 2011 software (Carl Zeiss). The ELYRA PS.1 system was calibrated using fluorescent beads (40 nm), yielding a lateral resolution of <80 nm and an axial resolution of ~275 nm. The SIM module sets the brightness levels differently for each image, so the images were lastly normalized in brightness levels to be comparable.

#### Confocal microscopy of RCPs in solution

We flowed the RCPs generated in solution through a microfluidic channel with a flow rate of 5 nl min^−1^ and simultaneously imaged the RCPs using a Zeiss LSM510 meta confocal laser scanning microscope equipped with a 40 × (1.3 NA) oil objective. We generated time line scan images using LSM (Zeiss) software, before analyzing them further using Matlab (Mathworks) using a procedure previously described in detail^9^.

### Image analysis

#### Quantification of RCP intensities and signal-to-noise ratios

Starting with image stacks of 18 or 16 z-levels we computed a pixelwise maximum (Fig. 2a,b) and a pixelwise minimum intensity projection. We subtracted the minimum intensity projection from each corresponding maximum intensity projection (Fig. 2c,d). We used an intensity threshold, automatically found by taking the mode of the histogram of the image, which is assumed to be the background of the image, and setting the threshold at twice that. Only pixels between 2% and 98% of the intensity range were used in these calculations. This is a method called “Background” in CellProfiler. We computed the signal-to-noise ratio of individual RCPs as the ratio of the maximum signal intensity to the standard deviation of the intensities of the local neighboring pixels. Measurements of signal-to-noise ratio can become incorrect due to neighboring signals interfering with the measurement of local background of an observed signal. We therefore excluded clustered RCPs, based on size and shape by removing detected objects larger than 40 pixels or with a circularity value below 0.5 (1 is a perfectly circular disk). We thereafter identified the centers of the remaining RCPs and defined each local signal as the maximum pixel intensity within a disk with fixed radius, centered at the RCP. We defined the neighborhood of an RCP as a circular band of fixed radius around the center of each RCP. In order to get a good statistical basis we ensured that each neighborhood was unique for the corresponding RCP. We did this by excluding RCPs that had overlapping neighborhoods (or neighborhoods overlapping with other RCPs). The remaining RCPs, constituting approximately 25% of the total detected RCPs, are shown as gray disks surrounded by a white neighborhood ring in (Fig. 2e,f). We finally calculated the ratio *r* of the maximum intensity within each inner (gray) disk to the standard deviation of all the pixels in the corresponding surrounding (white) disk. We used disks of inner-radius 3 and outer radius 7 (i.e., radii (3,7)pixels for signal and background respectively (Fig. 2h). In Supplementary Table S1 the same analysis was performed with inner-radius of 5 and outer-radius of 12 pixels. We converted the signal-to-noise ratio (SNR) to dB by SNR = 20 × log_10_(*r*). All analysis steps were implemented in CellProfiler^10^.

#### Proximity between signals, and signal co-localization

For analyzing signal proximity and co-localization (i.e., measuring the frequency of the event that two neighboring signals have the same color and the quantification of the signal overlap respectively), we performed a maximum intensity projection on each of the two color channels of an 18-level z-stack. The images for both the color channels FITC and Cy3 were filtered with a Gaussian kernel with a standard deviation equal to the length of a single pixel. The background was removed by subtraction of the image median. We localized the signals as local maxima. For measuring the signal proximity, we identified the closest neighbor (i.e. in either of the two color channels) of each signal by computing the Euclidean norm. Thereafter, we compared the colors of each signal and determined the frequency of the occurrence of signal pairs with same color as the ratio of the number of same color pairs to the sum of both same color and different color pairs. For co-localization analysis, the images for both the color channels were Gaussian filtered and binarized to signal and background with the same fixed threshold. The degree of the signal co-localization across the two color channels was measured as the ratio of the intersection of foreground pixels (i.e., part of the RCPs) in the two color channels to the union of the foreground pixels in both the channels. The analysis was performed using Matlab (The MathWorks, Inc.) and ImageJ^11^.

For signal co-localization analysis of RCPs in solution, we acquired confocal images of the RCPs and analyzed them as described previously^9^. Briefly, we analyzed the images using Matlab (Mathworks), starting with identifying RCPs using an intensity threshold of 35 photomultiplier tube values and a size threshold of 2-30 pixels. We measured the photomultiplier tube values in both Cy3 and Cy5 channels for all individual RCPs, before we determined the number of Cy3 positive, Cy5 positive and Cy3 + Cy5 positive RCPs. Lastly, we plotted the fraction of double labeled RCPs in the three different groups of compaction oligonucleotide concentrations (Fig. 3e).

#### Statistical tests

We used IBM SPSS Statistics for Macintosh release 23.0.0 (2014; IBM Corp.) for statistical analyses. All datasets were checked for normality of their distribution (one sample Kolmorov Smirnov test). If datasets were normally distributed we used a two-tailed t-test. If the datasets were non-normally distributed we used Mann-Whitney U tests. For RCA in solution with confocal image acquisition we performed an ANOVA with an LSD post hoc test. P values below 0.05 were considered to be statistically significant.

## Additional Information

**How to cite this article**: Clausson, C.-M. *et al.* Compaction of rolling circle amplification products increases signal integrity and signal-to-noise ratio. *Sci. Rep.* **5**, 12317; doi: 10.1038/srep12317 (2015).

## Supplementary Material

Supplementary Information

## Figures and Tables

**Table 1:** Oligonucleotides used for RCA and in *in situ* PLA.

**Oligonucleotide**	**Description**	**Vendor**	**DNA sequence**
A	Detection oligo Texas Red	Integrated DNA Technology	5' – CAGTGAATGCGAGTCCGTCTZZZZ – 3'
B	Compaction oligo	Integrated DNA Technology	5' – AGAGAGTAGTACAGCAGCCGTAAAAGAGAGTAGTACAGCAGCCGTZZZ^b^ – 3'
C^a^	Long circularization oligo	Integrated DNA Technology	5' – CTATTAGCGTCCAGTGAATGCGAGTCCGTCTA AGAGAGTAGTACAGCAGCCGTCAAGAGTGTCTA – 3'
D^a^	Short circularization oligo	Integrated DNA Technology	5' – GTTCTGTCATATTTAAGCGTCTTAA – 3'
E	Biotinylated RCA template	Eurogentech	5' – biotin-AAAAAAAAAATATGACAGAACTAGACACTCTT – 3'
F^a^	Long circularization oligo for Cy3	Integrated DNA Technology	5' – CTATTAGCGTCAAGAGAGTAGTACAGCAGCCGTATCAGTG AATGCGAGTCCGTCTAACTAGTGCTGGATGATCGTCCAAGAGT GTCTA – 3'
G^a^	Long circularization oligo for FITC and Cy5	Integrated DNA Technology	5' – CTATTAGCGTCAAGAGAGTAGTACAGCAGCCGTATCAGTG AATGCGAGTCCGTCTAAAGCGATCTGCGAGACCGTATAAGAGT GTCTA – 3'
H	Ligation template	Biomers	5' – GACGCTAATAGTTAAGACGCTTZZZ^b^ – 3'
I	Detection oligo Cy3	Integrated DNA Technology	5' – Cy3-CTAGTGCTGGATGATCGTCCZZZZ^b^ – 3'
J	Detection oligo FITC	Integrated DNA Technology	5' – FITC-AGCGATCTGCGAGACCGTATZZZZ^b^– 3'
K^a^	Padlock probe	Integrated DNA Technology	5' – GTTCTGTCATACAGTGAATGCGAGTCCGTCTAA GAGAGTAGTACAGCAGCCGTCAAGAGTGTCTA – 3'
L	Detection oligo Alexa 488	Integrated DNA Technology	5' – Alexa488-AAAAAACAGTGAATGCGAGTCCGTCTZZZZ^b^ – 3'
M	Ligation template	Integrated DNA Technology	5' – biotin-CTCTCTCTCTCTCTCTCTCTGTTCACGCTCACCGT GCCCAGTGAGCGAGGACTGCAGCGTAGACG – 3'
N^a^	Padlock probe	Integrated DNA Technology	5' – CACTGGGCACGGTGAGTGTATGCAGCTCCTC AGTAATAGTGTCTTACAAATCAGTCATACGAGCGCCGCTGCA GTCCTCGCT – 3'
O	Ligation template	Integrated DNA Technology	5' – GACGCTAATAGTAGACACTCTT – 3'
P	Detection oligo Cy5	Integrated DNA Technology	5' – Cy5-AGCGATCTGCGAGACCGTATZZZZ^b^ – 3'
Q	Detection oligo Alexa 642	Integrated DNA Technology	5'– Alexa642-AGCGATCTGCGAGACCGTATZZZ^b^ – 3´

^a^The oligonucleotide was phosphorylated at a concentration of 2.5 mM prior to use in a buffer containing 1 mM ATP (Thermo Scientific), 1x Reaction buffer A (Thermo Scientific) and 1 U/μl T4 Polynucleotide Kinase (EK0031; Thermo Scientific) for 30 min at 37 °C. The kinase was then heat inactivated at 65 °C for 15 min.

^b^Z represents 2’O-methyl-RNA Uracil.

## References

[b1] Liu D., Daubendiek S. L., Zillman M. A., Ryan K. & Kool E. T. Rolling Circle DNA Synthesis: Small Circular Oligonucleotides as Efficient Templates for DNA Polymerases. J Am Chem Soc 118, 1587–1594, 10.1021/ja952786k (1996). 20830216 PMC2935701

[b2] Schweitzer B. *et al.* Immunoassays with rolling circle DNA amplification: a versatile platform for ultrasensitive antigen detection. Proc Natl Acad Sci USA 97, 10113–10119, 10.1073/pnas.170237197 (2000). 10954739 PMC27732

[b3] Soderberg O. *et al.* Direct observation of individual endogenous protein complexes *in situ* by proximity ligation. Nature methods 3, 995–1000, 10.1038/nmeth947 (2006). 17072308

[b4] Larsson C., Grundberg I., Soderberg O. & Nilsson M. *In situ* detection and genotyping of individual mRNA molecules. Nature methods 7, 395–397, 10.1038/nmeth.1448 nmeth.1448 (2010). 20383134

[b5] Larsson C. *et al.* *In situ* genotyping individual DNA molecules by target-primed rolling-circle amplification of padlock probes. Nature methods 1, 227–232, 10.1038/nmeth723 (2004). 15782198

[b6] Dorfman K. D., King S. B., Olson D. W., Thomas J. D. & Tree D. R. Beyond gel electrophoresis: microfluidic separations, fluorescence burst analysis, and DNA stretching. Chem Rev 113, 2584–2667, 10.1021/cr3002142 (2013). 23140825 PMC3595390

[b7] Clausson C. M. *et al.* Increasing the dynamic range of *in situ* PLA. Nature methods 8, 892–893, 10.1038/nmeth (2011). 22036742

[b8] Gustafsson M. G. L. *et al.* Three-Dimensional Resolution Doubling in Wide-Field Fluorescence Microscopy by Structured Illumination. Biophysical Journal 94, 4957–4970, 10.1529/biophysj (2008). 18326650 PMC2397368

[b9] Jarvius J. *et al.* Digital quantification using amplified single-molecule detection. Nature methods 3, 725–727, 10.1038/nmeth916 (2006). 16929318

[b10] Kamentsky L. *et al.* Improved structure, function, and compatibility for CellProfiler: modular high-throughput image analysis software. Bioinformatics 8, 1179–1180, 10.1093/bioinformatics/btr095 (2011). 21349861 PMC3072555

[b11] Schindelin J. *et al.* Fiji: an open-source platform for biological-image analysis. Nat Meth 9, 676–682, 10.1038/nmeth.2019 (2012). PMC3855844 22743772

