# Supplementary material for: Compaction of rolling circle amplification products increases signal integrity and signal-to-noise ratio
Source: Sci Rep. 2015 Jul 23;5:12317. doi: 10.1038/srep12317 (PMC4511876; doi:10.1038/srep12317)
Supplement: Supplementary Information [file srep12317-s1.pdf]

# Compaction of rolling circle amplification products increases signal integrity and signal-to-noise ratio

Carl-Magnus Clausson[1*], Linda Arngården[1*], Omer Ishaq[2], Axel Klaesson[1], Malte Kühnemund[1], Karin Grannas[1], Björn Koos[1], Xiaoyan Qian[3], Petter Ranefall[2],Tomasz Krzywkowski[3], Hjalmar Brismar[4], Mats Nilsson[1,3], Carolina Wählby[2] & Ola Söderberg[1]

## Supplementary information

**Supplementary Table S1**: Although absolute values of intensity (a) and SNR (b) are affected by changing the disk radii (from 3 pixel inner radius and 7 pixel outer radius to 5 pixel inner radius and 12 pixel outer radius) the outcome of the three replicates, named No1-3 (i.e. compacted RCPs are brighter and have a higher SNR than regular RCPs) is not affected.

**a**

| Replicate | Average, compacted RCP (a.u.) | SD, compacted RCP (a.u.) | Average, regular RCP (a.u.) | SD, regular RCP (a.u.) | P value (Mann Whitney U) |
|---|---|---|---|---|---|
| No 1 (3,7) | 21.9 | 11.8 | 11.4 | 6.3 | p<0.001 |
| No 1(5,12) | 23.1 | 11.8 | 13.3 | 7.8 | p<0.001 |
| No 2(3,7) | 20.7 | 11.4 | 10.5 | 5.6 | p<0.001 |
| No 2(5,12) | 22.3 | 11.4 | 13.1 | 7.7 | p<0.001 |
| No 3(3,7) | 20.2 | 10.7 | 11.2 | 6.2 | p<0.001 |
| No 3(5,12) | 23.1 | 11.8 | 13.3 | 7.8 | p<0.001 |

**b**

| Replicate | Average, compacted RCP (dB) | SD, compacted RCP (dB) | Average, regular RCP (dB) | SD, regular RCP (dB) | P value (Mann Whitney U) |
|---|---|---|---|---|---|
| No 1 (3,7) | 14.8 | 1.9 | 11.2 | 3.0 | p<0.001 |
| No 1(5,12) | 18.0 | 2.2 | 12.7 | 3.9 | p<0.001 |
| No 2(3,7) | 14.6 | 2.0 | 10.6 | 3.0 | p<0.001 |
| No 2(5,12) | 17.5 | 2.7 | 11.6 | 3.8 | p<0.001 |
| No 3(3,7) | 14.5 | 2.0 | 11.0 | 3.1 | p<0.001 |
| No 3(5,12) | 17.4 | 2.6 | 12.5 | 4.0 | p<0.001 |
